# Highly Sensitive Lateral Flow Immunodetection of the Insecticide Imidacloprid in Fruits and Berries Reached by Indirect Antibody–Label Coupling

**DOI:** 10.3390/foods14010025

**Published:** 2024-12-25

**Authors:** Lyubov V. Barshevskaya, Elena A. Zvereva, Anatoly V. Zherdev, Boris B. Dzantiev

**Affiliations:** A.N. Bach Institute of Biochemistry, Research Center of Biotechnology of the Russian Academy of Sciences, Leninsky Prospect 33, 119071 Moscow, Russia; lyubov.barshevskaya@yandex.ru (L.V.B.); zverevaea@yandex.ru (E.A.Z.); zherdev@inbi.ras.ru (A.V.Z.)

**Keywords:** immunochromatographic assay, colloidal gold, neonicotinoids, food safety control, squeezed juices

## Abstract

A highly sensitive lateral flow immunoassay (LFIA) for imidacloprid, a widely used neonicotinoid insecticide, has been developed. The LFIA realizes the indirect coupling of anti-imidacloprid antibodies and gold nanoparticle (GNP) labels directly in the course of the assay. For this purpose, the common GNPs conjugate with anti-imidacloprid antibodies and are changed into a combination of non-modified, anti-imidacloprid antibodies, and the GNPs conjugate with anti-species antibodies. The given approach provides the possibility of selecting independent concentrations of GNPs and anti-imidacloprid antibodies to obtain the influence of minimal imidacloprid concentrations in the samples on the formation of detected, labeled immune complexes. A comparative study of imidacloprid LFIAs with common and indirect antibody–label coupling was implemented. The second variant reduced the limit of detection (LOD) of imidacloprid 20 times, reaching 0.2 ng/mL and 0.002 ng/mL for visual and instrumental detection, respectively, thus surpassing the existing LFIAs for imidacloprid. The developed highly sensitive LFIA was tested for imidacloprid detection in freshly squeezed fruits and berries without any additional sample preparation. The imidacloprids revealed were in the range of 75–97% for grape, 75–85% for orange, and 86–97% for apple samples. The time of the testing was 15 min.

## 1. Introduction

Control of pesticide residues in food stuffs is of great importance for the efficient protection of human health [1,2]. The extended use of neonicotinoid insecticides in recent decades makes the inclusion of these compounds a priority of this control. Although neonicotinoids are positioned as a new generation of less dangerous insecticides, a lot of information has been accumulated about their negative effects, which affect both the regulations concerning their use and the requirements with regards to their levels in foods. Neonicotinoids have caused the recent worldwide crisis, with mass deaths of bees [3,4]. In this regard, three intensively produced neonicotinoids, imidacloprid, clothianidin, and thiamethoxam, have been banned in the EU for outdoor uses, though they are continuing to be applied in permanent greenhouses and for other agricultural needs [5]. However, the risks of contamination of plant-based food products by neonicotinoids on the global market still seem to be very important [6]. Imidacloprid is of particular interest among neonicotinoids, due to the large volumes of its production and use, including illegal use. Despite the ban on the outside use of imidacloprid in the EU, the application of imidacloprid is widespread. Some countries continue to apply it in case of emergency use, which complicates imidacloprid application control [7,8].

Imidacloprid causes numerous negative effects on the environment [9,10,11], animals, and humans [12,13]. Consumption of imidacloprid-contaminated food can induce disorders of the endocrine, reproductive, and other systems of organisms [14,15,16]. Due to the reasons described above, regulations concerning contaminations of food by neonicotinoids are becoming more stringent [17], leading to the need for their sensitive detection.

The existing variety of analytical methods for imidacloprid includes chromatographic (liquid and gas chromatography, including ones with mass spectrometric detection) [18,19,20] and immunochemical (mainly microplate immunoenzyme assays) ones [21,22]. Although most of them are characterized by high sensitivity, accuracy, and specificity, the widespread application of these methods is hampered by the need for expensive equipment, qualified personnel, and complex sample preparation and analysis, resulting in a long wait for results, with rapid decision-making concerning the tested products being impossible [23,24].

Therefore, rapid methods that allow an analysis to be carried out directly at the sites of the study are in demand. Very successful solutions that meet these requirements are immunochromatographic test systems, which provide easy results that can be obtained within 10–20 min, without special equipment and trained personnel [25]. LFIA (lateral flow immunoassay) has found wide applications in a large number of areas: medicine [26,27], veterinary [28,29], agriculture [30], environmental monitoring [31], food quality control [32,33,34], etc. However, traditional LFIAs have certain limitations, first of all caused by the insufficient sensitivity of their common formats [35,36]. To overcome them, various approaches are proposed, such as new nanoparticle labels [37,38,39] and the involvement of additional reactions enhancing the detected signals [40,41,42,43]. However, these approaches are associated with the search for new synthetic techniques and their adoption of scaled manufacturing, complicating LFIA, due to the need for additional operator actions and new detection tools.

Therefore, solutions that reduce the detection limit within the framework of using standard LFIA components seem preferable for rapid improvements in widely used test systems. Note that the immunodetection of low-molecular-weight compounds is commonly realized by a competitive scheme. It is based on the competition between the antigen in the tested sample and in the immobilized hapten–protein conjugate on the test strip’s membrane for binding specific antibodies labeled with nanoparticles [44]. An alternate approach for the competitive LFIA with the indirect labeling of specific antibodies was proposed in our studies [45]. Namely, the conjugate of nanoparticles with specific antibodies is replaced by a combination of nanoparticle-labeled anti-species antibodies and free specific antibodies. As a result of such separation, the amount of the specific antibodies can be reduced with the stored high intensity of coloration, thus leading to a lower detection limit. The effectiveness of this approach has been demonstrated for several LFIAs [46,47,48,49].

The presented study considers the application of indirect antibody–label coupling in the LFIA of imidacloprid, testing its advantages in comparison with the common LFIA. The novelty of this study consists of the applicability of the proposed approach for the detection of imidacloprid for the first time. The efficiency of this approach varies greatly from several times to hundreds of times. Therefore, the evaluation of the proposed method for the detection of new analytes in new matrices is in demand and of great scientific interest. The efficiency of the developed assay is confirmed by its application to the testing of freshly squeezed grape, orange, and apple juices.

## 2. Materials and Methods

### 2.1. Reagents and Materials

Reagents used in the work included goat anti-mouse (anti-species) antibodies (Imtek, Moscow, Russia), chloroauric acid, imidacloprid, Tween-20, sodium citrate, sodium azide (Sigma-Aldrich, St. Louis, MO, USA), and bovine serum albumin (BSA) (Boval Biosolutions, Cleburne, TX, USA). Anti-imidacloprid monoclonal antibodies and the imidacloprid–BSA conjugate were from Creative Diagnostics (Shirley, NY, USA). Other salts, acids, and alkalis were of analytical or chemical grade. All syntheses were performed using water deionized by the Simplicity system (Millipore, Burlington, MA, USA).

Compounds for assembling test strips included the CNPC SS-12 working nitrocellulose membrane, AP-045 adsorption membrane, and L-P25 plastic support (Advanced Microdevices, Ambala Cantt, Haryana, India).

### 2.2. Gold Nanoparticles Synthesis

GNPs (gold nanoparticles) were obtained using the Frens method [50]. First, 100 milliliters of 0.01% HAuCl_4_ was heated to a boil. Then, 1.5 mL of 1% sodium citrate was added with stirring. After 15 min of boiling, the mixture was cooled and stored at 4 °C.

### 2.3. Transmission Electron Microscopy of GNPs

A CX-100 electron microscope (Jeol, Tokyo, Japan) was used at a magnification of 33,000 and an acceleration voltage of 80 kV, and the obtained images were processed by the Image Tool 3.0 program (UTHSCSA, San Antonio, TX, USA).

### 2.4. Conjugation of Anti-Imidacloprid and Anti-Species Antibodies with GNPs

The solution of the obtained GNPs was adjusted to pH 8.5 with the addition of 0.2 M K_2_CO_3_. The anti-imidacloprid or anti-species antibodies, after dialyzing them against a 10 mM Tris–HCl buffer, pH 8.0, were added to the GNPs at a final concentration of 10 μg/mL. Following 45 min of stirring, 10% BSA was added at a 40-fold dilution, and the mixture was agitated for 10 min at 60 rpm using an Intelli-Mixer RM-2S (Elmi, Riga, Latvia). Then, the GNPs were precipitated by centrifugation (15 min, 13,400× *g*, 4 °C). Following collection, the pellets were resuspended twice in 10 mM Tris, pH 8.5, containing 1% sucrose and 1% BSA (TBSA), and then centrifuged again. The final precipitate was redissolved in TBSA with 0.05% NaN_3_ and kept at 4 °C [51].

### 2.5. Production of Test Strips

To form the analytical zone, the imidacloprid–BSA conjugate (0.5 mg/mL in 50 mM PBS, pH 7.4) was applied on the nitrocellulose membrane using an IsoFlow dispenser (Imagene Technology, Lebanon, NH, USA). After the membrane was dried at 20–22 °C for 24 h, a composite of the working membrane, adsorption membrane, and plastic support was assembled, cut into strips with a 3.5 width using an Index Cutter-1 guillotine cutter (A-Point Technologies, Brea, CA, USA), and stored in a sealed package with silica gel at room temperature.

### 2.6. Implementation of Common LFIA

Imidacloprid 920–0.0013 ng/mL) was mixed with the GNP–anti-imidacloprid antibodies conjugate (OD_520_ = 0.5) in 20 µL PBS with 1% Tween-20 (PBST) in microplate wells and incubated for 3 min. The test strips with the immobilized imidacloprid–BSA conjugate were introduced into the wells in a vertical position. After 10 min, the test strips were removed, placed horizontally, and scanned.

### 2.7. Implementation of LFIA with Indirect Labeling

Imidacloprid (from 20 to 0.0013 ng/mL) was mixed with the GNP–goat anti-mouse antibodies conjugate (OD_520_ = 1.0) and anti-imidacloprid antibodies (50 ng/mL) in 20 µL of PBST or freshly squeezed grape, orange, or apple juices (dilution coefficient 10) in wells and left for 5 min. Then, the analysis was carried out as mentioned above.

### 2.8. Samples Preparation and Characterization

Grapes, oranges, and apples were purchased from the local grocery store. The oranges were preliminarily separated from the peel. The samples were ground using a kitchen homogenizer, centrifuged (6000× *g*, 5 min, 4 °C), and resulting supernatants were used as the preparations for the tested, freshly squeezed juices.

To confirm the applicability of the prepared samples as imidacloprid-free samples for the characterization of the developed LFIA, they were tested using the HPLC technique with mass spectrometric detection [52]. HPLC-MS/MS was carried out using an Agilent 1200 high-performance liquid chromatography system with an Agilent 6410B three-quadrupole mass spectrometer (Agilent Technologies, Santa Clara, CA, USA). The Agilent Poroshell120 EC-C18 column (4.6 × 50 mm, 2.7 µm) was applied for the separation at 40 °C in gradient elution mode. A 1% solution of formic acid (Merck, Rahway, NJ, USA) in water and acetonitrile (J.T. Baker, Phillipsburg, NJ, USA) were used to form the gradient, as specified in Table S1. The mobile phase flow rate was 0.4 mL/min. The injected sample volume was 10 μL. The mass spectrometric detection was characterized by the following parameters: source temperature 100 °C; desolvation gas temperature 350 °C; desolvation gas flow rate 12 L/min; and pressure nozzle needle 50 psi. The analytical signals recorded for imidacloprid in the multiple reaction monitoring (MRM) mode resulted in the following values: precursor ion, *m*/*z*, 256.1; product ion, *m*/*z*, 209.1 and 175.1; fragmentation potential, V, 90; and dissociation energy, V, 12 and 20, respectively.

The used HPLC-MS/MS method had the following validation parameters:

- Working range: 1000–0.1 ng/mL of imidacloprid (Figure S2);
- Used concentrations of models: 1000; 750; 500; 250; 100; 50; 25; 10, 1; and 0.1 ng/mL of imidacloprid;
- Approximation of concentration dependence for the method: y = 2.410442 × x^2^ + 6870.751152 × x;
- R2 value for the approximation: 0.99951227;
- Recovery: 97.8–113.1%.

Data characterizing the assay and its application for samples testing are given in the Supplementary Information (Table S1, Figures S1–S3).

### 2.9. Processing Test Strip Images and Calculating Assay Parameters

Following the LFIA, the test strips were scanned at a 600 dpi resolution using a Canon Lide 90 flatbed scanner (Canon, Tokyo, Japan). The Total Lab program (Nonlinear Dynamics, Newcastle upon Tyne, UK) was then used for analysis. For all of the data in the article, line coloring intensities were shown in the same relative units.

The dependences of coloring intensity in the analytical zone (y) on the antigen concentration in the sample (x) were approximated by a four-parameter sigmoid function using the Origin 9.0 software (OriginLab, Northampton, MA, USA):

Y = (a − b)/[1 + (x/c)d] + b,(1)

where a = maximal signal, b = minimal signal, c (or IC_50_) = the antigen concentration at which the decrease in y was 50% of its range of changes, and d = the slope of the approximating dependence at point c.

The antigen concentration causing the disappearance of the analytical zone coloration was considered the visual detection limit.

## 3. Results

### 3.1. Characterization of Gold Nanoparticles

**Figure 1:**
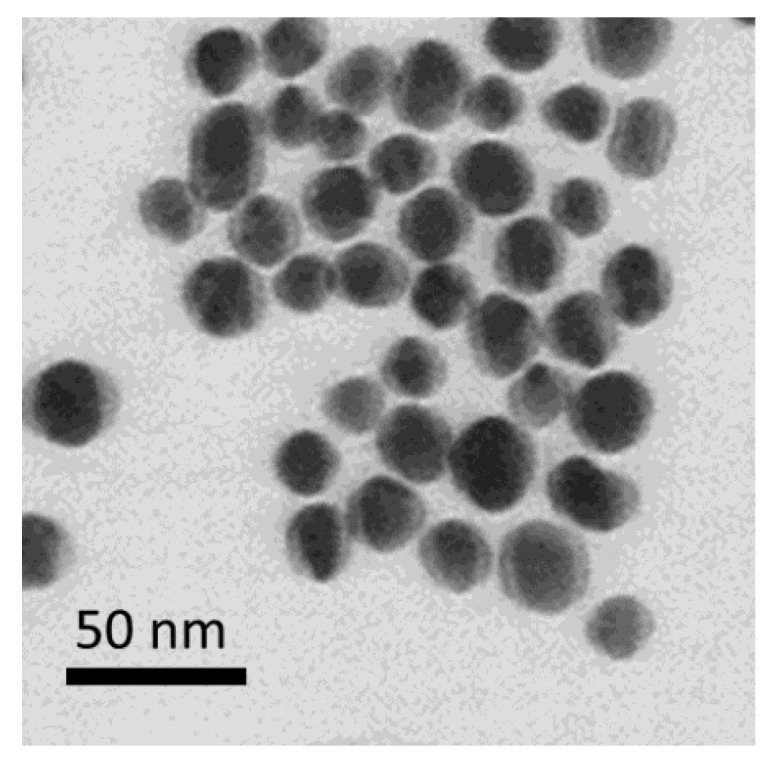
Transmission electron microscopy micrograph for GNPs.

GNPs were obtained by the Frens method [50] under the conditions that were chosen on the basis of the recommendations for the preferable use of GNPs, with diameters of 20–40 nm for the LFIA [53]. The average diameter of the nanoparticles was 20.83 ± 5.05 nm (n = 300), with a minimum value 10.43 nm and a maximum value of 27.67 nm, and the degree of ellipticity was 1.12 ± 0.08 (Figure 1).

### 3.2. Optimization of the Conditions for Common Competitive LFIA

**Figure 2:**
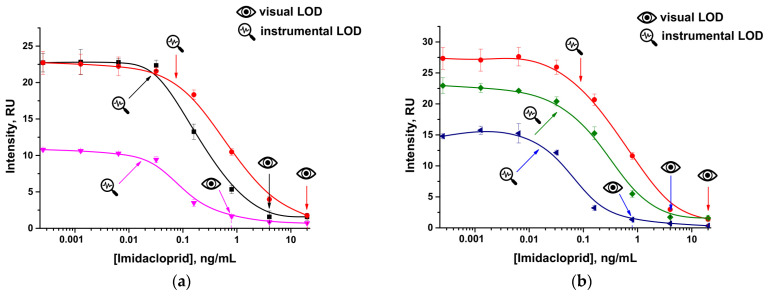
Concentration dependences for the common LFIA of imidacloprid under varied conditions: (**a**) for applied concentrations of the imidacloprid–BSA conjugate equal to 0.25 mg/mL (pink curve), 0.5 mg/mL (black curve), and 1.0 mg/mL (red curve); (**b**) for applied solutions of the GNP–anti-imidacloprid antibody conjugate with OD_520_ equal to 2.0 (red curve), 1.0 (green curve), and 0.5 (blue curve). All experiments were carried out in triplicate.

To enable the comparative evaluation of two approaches in the LFIA of imidacloprid, we selected their optimal conditions, starting from the common competitive scheme. For this purpose, the dependences of the detection limit of the test system on the concentration of the immobilized hapten–protein conjugate and on the concentration of the GNP–anti- imidacloprid antibody conjugate were established. Firstly, the concentration of the imidacloprid–BSA conjugate applied to the test strip was varied. As can be seen from the data obtained (Figure 2a), when the concentration of the conjugate decreased (1–0.25 mg/mL), the achieved instrumental (0.7–0.2 ng/mL) and visual (20–0.8 ng/mL) LOD (limit of detection) values decreased, but at the same time, the coloration intensity decreased (23–11 RU (relative units)). A conjugate concentration of 0.5 mg/mL was selected, which allowed the maintenance of a high intensity of the recorded coloration. Subsequently, the optimal concentration of the label’s conjugate was established (Figure 2b). A decrease in its concentration (optical density, OD_520_ 2.0–0.5) also led not only to the LOD shift (instrumental: 0.08–0.02 ng/mL; visual: 20–0.8 ng/mL) but also to a decrease in the coloration intensity (27–15 RU). The concentration of the GNP–anti-imidacloprid antibody conjugate was chosen to be OD_520_ 0.5.

**Figure 3:**
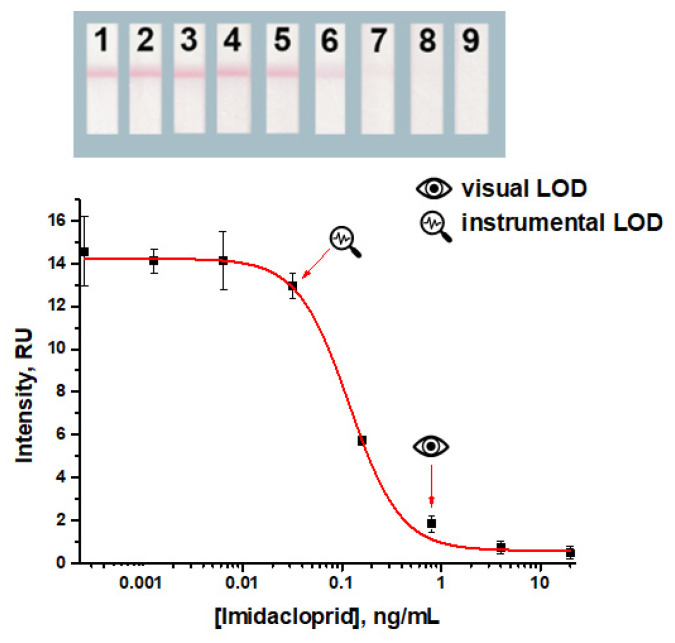
Concentration dependence for the common LFIA under the chosen conditions, namely applying imidacloprid–BSA from a solution with a concentration of 0.5 mg/mL and the GNP–anti-imidacloprid antibody conjugate from a solution with OD_520_ 0.5. The concentrations of imidacloprid in the samples for the test strips numbered as 1–9 on the insert were: 0.0; 0.0003; 0.001; 0.006; 0.03; 0.2; 0.8; 4; and 20 ng/mL, respectively. All experiments were carried out in triplicate.

Basing on the chosen parameters, imidacloprid detection was implemented. The obtained values of the visual and instrumental detection limits were 0.8 ng/mL and 0.03 ng/mL, respectively (Figure 3).

### 3.3. Optimization of the Conditions for LFIA Based on Indirect Labeling

In the indirect labeling scheme, the conjugate of nanoparticles with specific antibodies was replaced by free specific antibodies combined with a conjugate of nanoparticles with anti-species antibodies. This approach allowed the independent variation in the concentration of the marker and specific antibodies to simultaneously reach efficient competition and high coloration intensity. Additionally, the factor of non-productive binding in the common competitive LFIA, when the multivalent nanoparticle-specific antibody conjugate stored the ability for binding at the test strip, with a significant part of the specific antibodies on its surface blocked by the antigen in the sample, was excluded by the indirect labeling [52].

**Figure 4:**
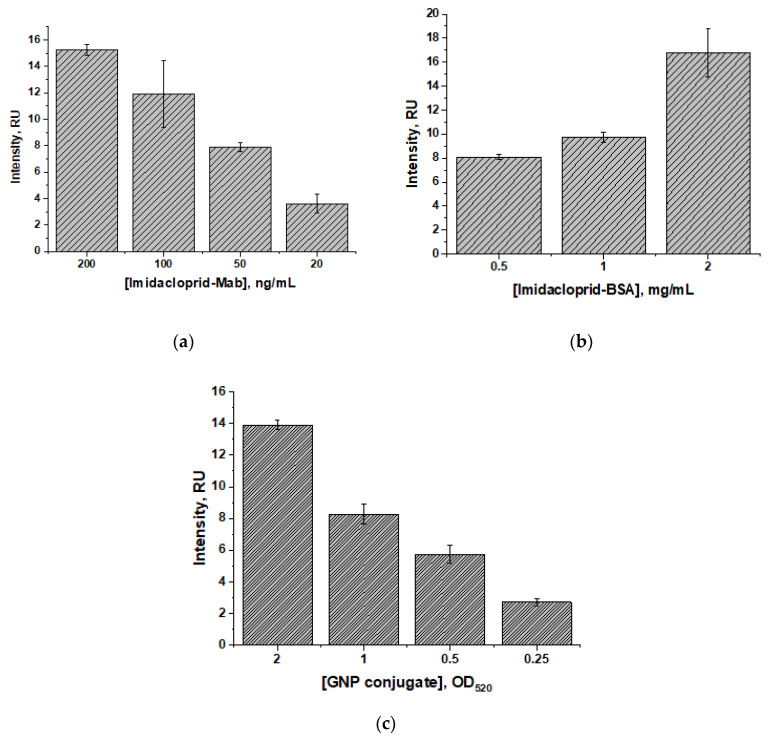
Dependences of the coloration intensity for the LFIA with indirect labeling on the concentrations of anti-imidacloprid antibodies (**a**), imidacloprid–BSA conjugate (**b**), and the GNP conjugate with anti-species antibodies (**c**). The LFIA was performed for solutions without imidacloprid. All experiments were carried out in triplicate.

To choose the conditions for the imidacloprid LFIA with indirect labeling, the dependences of the coloration intensity and the detection limit on such parameters as the concentration of the imidacloprid–BSA conjugate, the concentration of specific antibodies, and the concentration of the GNP conjugate with anti-species antibodies were established. Figure 4a shows that an increased concentration of antibodies against imidacloprid (20–200 ng/mL) increased the recorded coloration intensity (3.6–15 RU). A similar effect was observed when varying the concentration of the marker conjugate (Figure 4b), namely that its increase (OD_520_ 0.25–2) led to an increase in the coloration intensity (2.7–14 RU). In the case of the imidacloprid–BSA conjugate, varying its concentrations from 0.5 to 1 mg/mL did not lead to a significant difference in the recorded coloration, in contrast to using a higher concentration of 2 mg/mL (16.7 RU)—see Figure 4c. In general, as a result of increasing the concentrations of the test system components, the coloration intensity increased.

However, these changes also led to shifts in the LOD towards higher concentrations. As can be seen from Table 1, when using high concentrations of the test system components (2–1 mg/mL for the imidacloprid–BSA conjugate, 200–100 ng/mL for specific antibodies, and OD_520_ 2.0–1.0 for the marker conjugate), the LOD values also increased (visual: 0.2–20 ng/mL; instrumental: 0.002–0.09 ng/mL). In turn, the use of the lowest concentrations of the test system components (20 ng/mL for specific antibodies and OD_520_ 0.25 for the marker conjugate) reduced the visual LOD to 0.03 ng/mL and instrumental LOD to 0.0005 ng/mL. However, visual detection in this case became difficult (the coloration intensity was only 2 RU), which can reduce the reliability of conclusions. The registration of the instrumental LOD ensured the reproducibility and reliability of the analysis results.

Thus, the optimal condition for performing the imidacloprid LFIA with indirect labeling was chosen as a combination of concentrations of the test system components (50 ng/mL for specific antibodies, 0.5 mg/mL for the imidacloprid–BSA conjugate, and OD_520_ 1.0 for the marker conjugate), which lowered the LOD, with the simultaneous ability to reliably visually evaluate the assay results.

**Figure 5:**
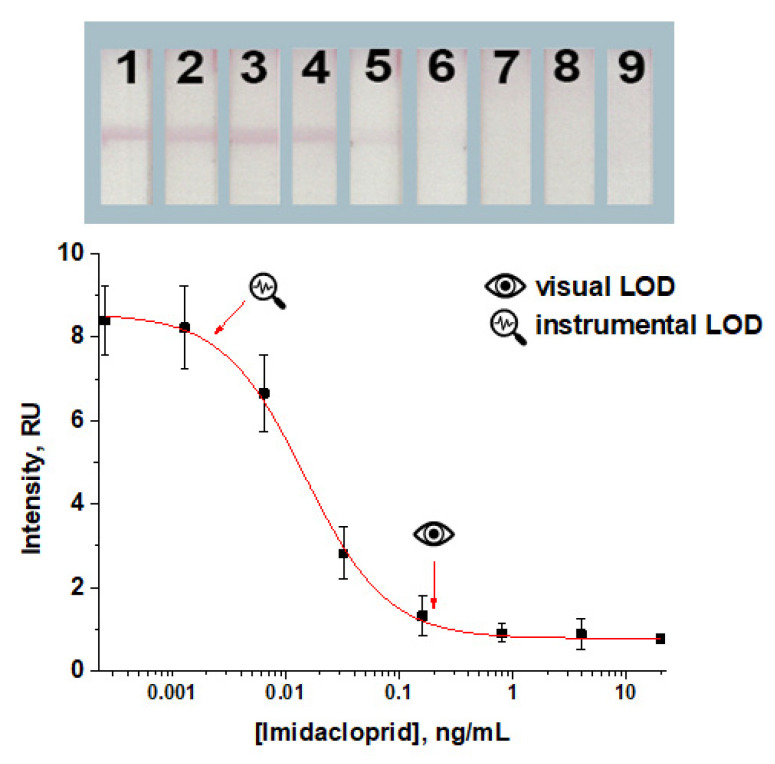
The LFIA for imidacloprid based on indirect labeling under the chosen conditions (50 ng/mL for anti-imidacloprid antibodies, 0.5 mg/mL for the applied imidacloprid–BSA conjugate, and OD_520_ 1.0 for the applied GNP–anti-species antibodies conjugate). The concentrations of imidacloprid in the samples for the test strips numbered as 1–9 on the insert were 0; 0.0003; 0.001; 0.006; 0.03; 0.2; 0.8; 4; and 20 ng/mL, respectively. All experiments were carried out in triplicate.

Based on the established conditions, the LFIA of imidacloprid with indirect labeling was performed (Figure 5). According to the results obtained, the visual and instrumental detection limits were 0.2 and 0.002 ng/mL, respectively.

### 3.4. Approbation of the Developed LFIA on Food Matrices

**Figure 6:**
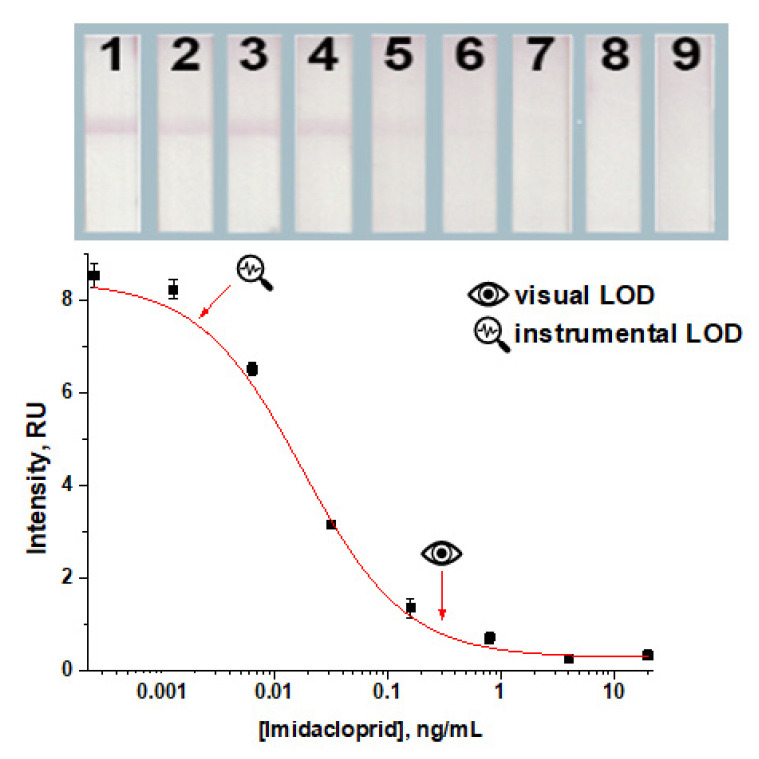
Example of the indirect LFIA for imidacloprid in squeezed grape juice. The concentrations of imidacloprid in the samples for the test strips numbered as 1–9 on the insert were 0.0; 0.0003; 0.001; 0.006; 0.03; 0.2; 0.8; 4; and 20 ng/mL, respectively. The equation of the approximating four-parametric sigmoidal fitting was y = 0.2754 + (8.4161 − 0.2754)/(1 + (x/0.0176)^0.9502^; its IC20–IC80 range was 0.004–0.076; R2 = 0.9919. All experiments were carried out in triplicate.

The proposed LFIA for imidacloprid based on indirect labeling was tested for freshly squeezed grape, orange, and apple juices diluted 10 times with PBST, containing imidacloprid in concentrations from 20 to 0.0003 ng/mL (Figure 6). The obtained visual detection limits for all three extracts were 0.2 ng/mL, and instrumental detection limits were 0.002 ng/mL (0.02 µg/kg), which is significantly lower than the maximal permissible levels for imidacloprid, according to the regulatory authorities of the European Union, Russia, China, Japan, Canada, and other countries and organizations [54]. For example, in the European Union, the MRLs of imidacloprid in grapes (700 µg/kg), oranges (900 µg/kg), and apples were equal to 700 µg/kg, 900 µg/kg, and 10 µg/kg, respectively [55]. The duration of the analysis was 15 min. The obtained results demonstrated the possibility of the effective application of the developed approach for the detection of small amounts of imidacloprid in food products. (The achieved values of the LOD were lower than the established MRL by at least 5 (for visual LOD) and 500 times (for instrumental LOD).

The estimated amount of monoclonal antibodies to imidacloprid in the proposed LFIA was equal to 1 ng per one test strip, while the amount of monoclonal antibodies per one test strip in the LFIA with direct labeling was equal to 20 ng/mL. Thus, the reduction in antibody consumption reached 20 times.

The testing of the grape, orange, and apple samples demonstrated that the revealing of imidacloprid was in the range of 75–97% (Table 2).

### 3.5. Selectivity of the Developed LFIA

**Figure 7:**
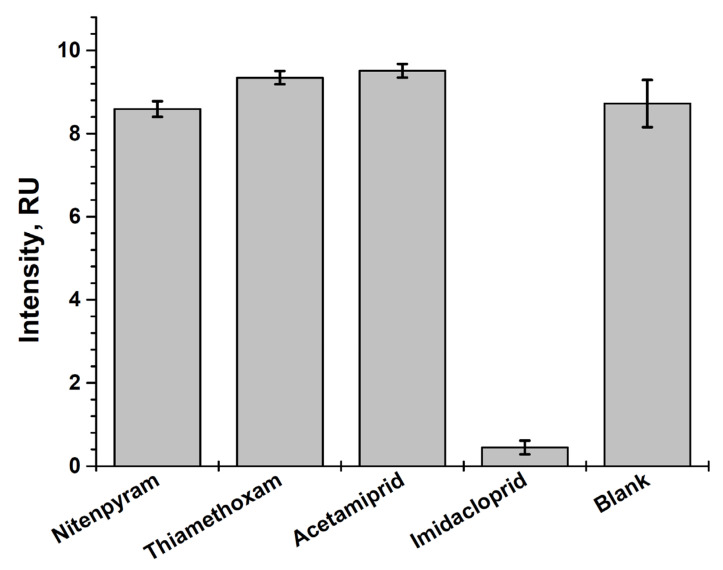
Selectivity testing of the developed LFIA for imidacloprid based on indirect labeling for different neonicotinoid insecticides.

To test the selectivity of the developed assay, three insecticides from the neonicotinoid class (acetamiprid, thiamethoxam, and nitenpyram) were studied under the chosen conditions. For this purpose, the insecticides were added to the sample at a concentration of 500 ng/mL, antibodies against imidacloprid at a concentration of 50 ng/mL, and a conjugate of GNPs with anti-species antibodies at OD_520_ 1.0. After incubation (5 min), a test strip was immersed in the resulting sample. According to the data obtained, during the interaction with non-target analytes (Figure 7), the coloration intensity in the analytical zone did not differ reliably from the blank values obtained in the absence of imidacloprid. When imidacloprid (100 ng/mL) was added, the coloration of the analytical zone was not observed.

Additionally, the absence of non-specific interactions of the immune reactants with widely used representatives of other groups of pesticides was confirmed by ELISA testing (Figure S4).

### 3.6. Comparison of the Developed LFIA with Other LFIAs of Imidacloprid

The developed LFIA with indirect labeling allowed the reduction of the LOD of imidacloprid by up to 20 times with the same immunoreactants as the common LFIA, which proved its competitive advantages in comparison with other LFIAs for imidacloprid (Table 3).

Considering the place of the proposed development among other methods of IMD detection, we noted that chromatographic methods (LC and GC, including their variants with mass spectrometric detection) and ELISA dominated among them. These methods can be implemented only in stationary laboratories using specialized equipment, i.e., they are not suitable for mass on-site testing. The limits of imidacloprid detection using chromatographic methods presented in recent works [63,64,65,66,67,68] varied from 0.02 to 40 ng/mL, depending on the protocols of the IMD extraction and concentration, as well as on the used method of its detection. ELISA methods [69,70] are characterized by less variability of detection limits, from 0.15 to 1.56 ng/mL. As we can see, the assay proposed in our work was competitive in comparison with alternative, more complex techniques, and in instrumental registration mode, it even ensured lower LODs of imidacloprid.

## 4. Conclusions

The LFIA based on indirect labeling with an effective reduction in the detection limit (20 times, compared to traditional immunochromatography) for the detection of imidacloprid was developed. The use of a conjugate of gold nanoparticles with anti-species antibodies allowed for an independent variation in the amount of marker and specific antibodies. This provided possibilities for a simultaneous, efficient competition process, with a lowered detection limit and high intensity of coloration for the reliable assessment of the assay results. The demonstrated effectiveness of the developed LFIA confirmed its potential to detect low concentrations of IMD in apples, oranges, and grapes within 15 min with visual and instrumental LODs at 2 µg/kg and 0.02 µg/kg, respectively. The reached LODs were much lower than the official maximal permissible levels for IMD in grapes (700 µg/kg), oranges (900 µg/kg), and apples (10 µg/kg). Therefore, the LFIA with indirect antibody labeling ensured the effective control of food quality and safety.

## Figures and Tables

**Table 1:** Dependence of visual and instrumental LODs for imidacloprid in LFIA with indirect labeling on concentrations of anti-imidacloprid antibodies, the imidacloprid–BSA conjugate, and the GNP–anti-species antibody conjugate.

Concentration	Visual LOD, ng/mL	Instrumental LOD, ng/mL
Anti-imidacloprid antibodies		
20 ng/mL	0.03	0.0007
50 ng/mL	0.2	0.002
100 ng/mL	20	0.07
200 ng/mL	20	0.09
Imidacloprid–BSA conjugate		
0.5 mg/mL	0.2	0.002
1 mg/mL	4	0.07
2.0 mg/mL	20	0.09
GNP–anti-species antibody conjugate		
0.25 OD_520_	0.03	0.0005
0.5 OD_520_	0.2	0.001
1.0 OD_520_	0.2	0.001
2.0 OD_520_	0.8	0.009

**Table 2:** Recoveries of imidacloprid in samples of juices for the LFIA with indirect labeling (n = 3).

Imidacloprid Added, pg/mL	Imidacloprid Detected, pg/mL	SD (n = 3), %	Recovery, %	RSD (n = 3), %
	Grape juice			
160	120.0	20.1	75	16.7
32	29.5	2.3	92	7.8
6.4	6.2	0.7	97	11.3
	Orange juice			
160	120.1	15.8	75	13.2
32	27.1	3.6	85	13.3
6.4	5.0	0.9	78	18
	Apple juice			
160	151.3	7.6	95	5
32	27.8	3.3	86	11.9
6.4	6.2	0.4	97	6.5

**Table 3:** Comparison of the LODs of LFIAs for imidacloprid in other reported studies.

Type of Label	LFIA Formats	Type of Detection	Tested Agricultural Products	Limit of Detection	Reference
GNP	Common LFIA	Colorimetric	Chinese leek	0.02 ng/mL (instrumental)	[56]
Time-resolved fluorescent nanobead	Common LFIA	Fluorescent	Chinese leek	0.02 ng/mL (instrumental)	[56]
GNP	Common LFIA	Colorimetric	Apple	20 ng/mL (visual)	[57]
GNP	Common LFIA	Colorimetric	Chinese cabbage	50 ng/mL (visual)	[58]
MoS2@Au@Ab	Common LFIA	Colorimetric	Tea	5 µg/mL (visual)	[59]
MoS2@Au@Ab	Common LFIA	Photothermal	Apple, cucumber	180 ng/mL (instrumental)	[60]
Quantum dots	Common LFIA	Fluorescent	Chinese cabbage	14.58 ng/mL (instrumental)	[61]
Scandum-Tetrakis (4-carboxyphenyl) porphyrin metal-organic framework nanocube	Common LFIA	Colorimetric	Tomato, corn, carrot and millet	1.5–3 ng/mL (visual)	[62]
GNP	LFIA with indirect labeling	Colorimetric	Grape, orange, and apple	0.2 ng/mL (visual)	This study
GNP	LFIA with indirect labeling	Colorimetric	Grape, orange, and apple	0.002 ng/mL (instrumental)	This study

## Data Availability

The original contributions presented in the study are included in the article/Supplementary Material, further inquiries can be directed to the corresponding author.

## Supplementary Materials

The following supporting information can be downloaded at https://www.mdpi.com/article/10.3390/foods14010025/s1, Table S1. Conditions of chromatographic detection; Figure S1. Chromatogram of the sample with imidacloprid at a concentration of 25 ng/mL; Figure S2. Linear calibration plot for imidacloprid; Figure S3. Example of chromatogram in grape juice (a) and orange juice (b); Figure S4. Selectivity testing of specific antibodies in ELISA. Black curve—paraquat; red curve—fipronil; blue curve—lambda-cyhalothrin; pink curve—chlorpyrifos; green curve—cyproconazole; orange curve—imidacloprid.

## References

[1] Fung F., Wang H.-S., Menon S. (2018). Food safety in the 21st century. Biomed. J..

[2] Garcia S.N., Osburn B.I., Jay-Russell M.T. (2020). One Health for food safety, food security, and sustainable food production. Front. Sustain. Food Syst..

[3] Fairbrother A., Purdy J., Anderson T., Fell R. (2014). Risks of neonicotinoid insecticides to honeybees. Environ. Toxicol. Chem..

[4] Sgolastra F., Medrzycki P., Bortolotti L., Maini S., Porrini C., Simon-Delso N., Bosch J. (2020). Bees and pesticide regulation: Lessons from the neonicotinoid experience. Biol. Conserv..

[5] Commission Implementing Regulation (EU) 2018/783 of 29 May 2018 Amending Implementing Regulation (EU) No 540/2011 as Regards the Conditions of Approval of the Active Substance Imidacloprid. https://eur-lex.europa.eu/legal-content/EN/TXT/PDF/?uri=CELEX:32018R0783.

[6] Simon-Delso N., Amaral-Rogers V., Belzunces L.P., Bonmatin J.-M., Chagnon M., Downs C., Furlan L., Gibbons D.W., Giorio C., Girolami V. (2015). Systemic insecticides (neonicotinoids and fipronil): Trends, uses, mode of action and metabolites. Environ. Sci. Pollut. Res. Int..

[7] Epstein Y., Chapron G., Verheggen F. (2022). What is an emergency? Neonicotinoids and emergency situations in plant protection in the EU. Ambio.

[8] Yu X., Pu H., Sun D.W. (2023). Developments in food neonicotinoids detection: Novel recognition strategies, advanced chemical sensing techniques, and recent applications. Crit. Rev. Food. Sci. Nutr..

[9] Morrissey C.A., Mineau P., Devries J.H., Sanchez-Bayo F., Liess M., Cavallaro M.C., Liber K. (2015). Neonicotinoid contamination of global surface waters and associated risk to aquatic invertebrates: A review. Environ. Int..

[10] Cycoń M., Markowicz A., Borymski S., Wójcik M., Piotrowska-Seget Z. (2013). Imidacloprid induces changes in the structure, genetic diversity and catabolic activity of soil microbial communities. J. Environ. Manag..

[11] Tišler T., Jemec A., Mozetič B., Trebše P. (2009). Hazard identification of imidacloprid to aquatic environment. Chemosphere.

[12] Nugnes R., Russo C., Orlo E., Lavorgna M., Isidori M. (2023). Imidacloprid: Comparative toxicity, DNA damage, ROS production and risk assessment for aquatic non-target organisms. Environ. Pollut..

[13] Duzguner V., Erdogan S. (2010). Acute oxidant and inflammatory effects of imidacloprid on the mammalian central nervous system and liver in rats. Pestic. Biochem. Physiol..

[14] Hafez E.M., Issa S.Y., Ai-Mazroua M., Ibrahim K.T., Rahman S.M.A. (2016). The neonicotinoid insecticide imidacloprid: A male reproductive system toxicity inducer-human and experimental study. Toxicol. Open Access.

[15] Imidacloprid: Human Health Draft Risk Assessment (2017). United States Environmental Protection Agency. https://downloads.regulations.gov/EPA-HQ-OPP-2008-0844-1235/content.pdf.

[16] Han W., Tian Y., Shen X. (2018). Human exposure to neonicotinoid insecticides and the evaluation of their potential toxicity: An overview. Chemosphere.

[17] Carrasco Cabrera L., Di Piazza G., Dujardin B., Marchese E., Medina Pastor P. (2024). The 2022 European Union report on pesticide residues in food. EFSA J..

[18] Wang Y., Fu Y., Wang Y., Lu Q., Ruan H., Luo J., Yang M. (2022). A comprehensive review on the pretreatment and detection methods of neonicotinoid insecticides in food and environmental samples. Food Chem. X.

[19] Chen S., Wang Y., Liu X., Ding L. (2023). Recent advances for imidacloprid detection based on functional nanomaterials. Chemosensors.

[20] Jiménez-López J., Llorent-Martínez E.J., Ortega-Barrales P., Ruiz-Medina A. (2020). Analysis of neonicotinoid pesticides in the agri-food sector: A critical assessment of the state of the art. Appl. Spectrosc. Rev..

[21] Reynoso E.C., Torres E., Bettazzi F., Palchetti I. (2019). Trends and perspectives in immunosensors for determination of currently-used pesticides: The case of glyphosate, organophosphates, and neonicotinoids. Biosensors.

[22] Zhou L., Yang J., Tao Z., Eremin S.A., Hua X., Wang M. (2020). Development of fluorescence polarization immunoassay for imidacloprid in environmental and agricultural samples. Front. Chem..

[23] Umapathi R., Park B., Sonwal S., Rani G.M., Cho Y., Huh Y.S. (2022). Advances in optical-sensing strategies for the on-site detection of pesticides in agricultural foods. Trends Food Sci. Technol..

[24] Raeisossadati M.J., Danesh N.M., Borna F., Gholamzad M., Ramezani M., Abnous K., Taghdisi S.M. (2016). Lateral flow based immunobiosensors for detection of food contaminants. Biosens. Bioelectron..

[25] Jiang N., Tansukawat N.D., Gonzalez-Macia L., Ates H.C., Dincer C., Güder F., Tasoglu S., Yetisen A.K. (2021). Low-cost optical assays for point-of-care diagnosis in resource-limited settings. ACS Sens..

[26] Wang C., Liu M., Wang Z., Li S., Deng Y., He N. (2021). Point-of-care diagnostics for infectious diseases: From methods to devices. Nano Today.

[27] Gumus E., Bingol H., Zor E. (2023). Lateral flow assays for detection of disease biomarkers. J. Pharm. Biomed. Anal..

[28] Amin N., Almasi A., Ozer T., Henry S.C., Hosseinzadeh L., Keshavarzi Z. (2023). Recent advances of optical biosensors in veterinary medicine: Moving towards the point of care applications. Curr. Top. Med. Chem..

[29] Hobbs E.C., Colling A., Gurung R.B., Allen J. (2021). The potential of diagnostic point-of-care tests (POCTs) for infectious and zoonotic animal diseases in developing countries: Technical, regulatory and sociocultural considerations. Transbound. Emerg. Dis..

[30] Mondal R., Dam P., Chakraborty J., Paret M.L., Katı A., Altuntas S., Sarkar R., Ghorai S., Gangopadhyay D., Mandal A.K. (2022). Potential of nanobiosensor in sustainable agriculture: The state-of-art. Heliyon.

[31] Nnachi R.C., Sui N., Ke B., Luo Z., Bhalla N., He D., Yang Z. (2022). Biosensors for rapid detection of bacterial pathogens in water, food and environment. Environ. Int..

[32] Anfossi L., Baggiani C., Giovannoli C., D’Arco G., Giraudi G. (2013). Lateral-flow immunoassays for mycotoxins and phycotoxins: A review. Anal. Bioanal. Chem..

[33] Zhou S., Xu L., Kuang H., Xiao J., Xu C. (2020). Immunoassays for rapid mycotoxin detection: State of the art. Analyst.

[34] Guan T., Xu Z., Wang J., Liu Y., Shen X., Li X., Sun Y., Lei H. (2022). Multiplex optical bioassays for food safety analysis: Toward on-site detection. Compr. Rev. Food Sci. Food Saf..

[35] Gordon J., Michel G. (2008). Analytical sensitivity limits for lateral flow immunoassays. Clin. Chem..

[36] Jackson T.M., Ekins R.P. (1986). Theoretical limitations on immunoassay sensitivity. Current practice and potential advantages of fluorescent Eu^3+^ chelates as non-radioisotopic tracers. J. Immunol. Methods.

[37] He W., Wang M., Cheng P., Liu Y., You M. (2024). Recent advances of upconversion nanoparticles-based lateral flow assays for point-of-care testing. Trends Anal. Chem..

[38] Wang Z., Zhao J., Xu X., Guo L., Xu L., Sun M., Hu S., Kuang H., Xu C., Li A. (2022). An overview for the nanoparticles-based quantitative lateral flow assay. Small Methods.

[39] Xiao X., Hu S., Lai X., Peng J., Lai W. (2021). Developmental trend of immunoassays for monitoring hazards in food samples: A review. Trends Food Sci. Technol..

[40] Calabria D., Calabretta M.M., Zangheri M., Marchegiani E., Trozzi I., Guardigli M., Michelini E., Di Nardo F., Anfossi L., Baggiani C. (2021). Recent advancements in enzyme-based lateral flow immunoassays. Sensors.

[41] Panferov V.G., Safenkova I.V., Varitsev Y.A., Zherdev A.V., Dzantiev B.B. (2018). Enhancement of lateral flow immunoassay by alkaline phosphatase: A simple and highly sensitive test for potato virus X. Microchim. Acta.

[42] Rodríguez M.O., Covián L.B., García A.C., Blanco-López M.C. (2016). Silver and gold enhancement methods for lateral flow immunoassays. Talanta.

[43] Yin X., Liu S., Kukkar D., Wang J., Zhang D., Kim K.-H. (2024). Performance enhancement of the lateral flow immunoassay by use of composite nanoparticles as signal labels. Trends Anal. Chem..

[44] Posthuma-Trumpie G.A., Korf J., van Amerongen A. (2009). Lateral flow (immuno)assay: Its strengths, weaknesses, opportunities and threats. A literature survey. Anal. Bioanal. Chem..

[45] Urusov A.E., Petrakova A.V., Zherdev A.V., Dzantiev B.B. (2016). “Multistage in one touch” design with a universal labelling conjugate for high-sensitive lateral flow immunoassays. Biosens. Bioelectron..

[46] Zvereva E.A., Hendrickson O.D., Solopova O.N., Zherdev A.V., Sveshnikov P.G., Dzantiev B.B. (2022). Triple immunochromatographic test system for detection of priority aquatic toxins in water and fish. Anal. Bioanal. Chem..

[47] Bartosh A.V., Urusov A.E., Petrakova A.V., Kuang H., Zherdev A.V., Dzantiev B.B. (2020). Highly sensitive lateral flow test with indirect labelling for zearalenone in baby food. Food Agr. Immunol..

[48] Li G., Xu L., Wu W., Wang D., Jiang J., Chen X., Zhang W., Poapolathep S., Poapolathep A., Zhang Z. (2019). On-site ultrasensitive detection paper for multiclass chemical contaminants via universal bridge-antibody labeling: Mycotoxin and illegal additives in milk as an example. Anal. Chem..

[49] Majdinasab M., Zareian M., Zhang Q., Li P. (2019). Development of a new format of competitive immunochromatographic assay using secondary antibody–europium nanoparticle conjugates for ultrasensitive and quantitative determination of ochratoxin A. Food Chem..

[50] Frens G. (1973). Controlled nucleation for the regulation of the particle size in monodisperse gold suspensions. Nat. Phys. Sci..

[51] Sotnikov D.V., Barshevskaya L.V., Zherdev A.V., Dzantiev B.B. (2023). Enhanced lateral flow immunoassay with double competition and two kinds of nanoparticles conjugates for control of insecticide imidacloprid in honey. Biosensors.

[52] Lin L., Peng Z., Yang C.L., Wang M.Y., Zha Y.B., Liu L.L., Zeng S.D. (2013). Determination of imidacloprid, carbendazim and thiabendazole residues in vegetables and fruits by HPLC. Adv. Mater. Res..

[53] Parolo C., Sena-Torralba A., Bergua J.F., Calucho E., Fuentes-Chust C., Hu L., Rivas L., Álvarez-Diduk R., Nguyen E.P., Cinti S. (2020). Tutorial: Design and fabrication of nanoparticle-based lateral-flow immunoassays. Nat. Protoc..

[54] Zhou J., Dong C., An W., Zhao Q., Zhang Y., Li Z., Jiao B. (2021). Dissipation of imidacloprid and its metabolites in Chinese prickly ash (Zanthoxylum) and their dietary risk assessment. Ecotoxicol. Environ. Saf..

[55] Commission Regulation (EU) 2021/1881 of 26 October 2021 Amending Annexes II and III to Regulation (EC) No 396/2005 of the European Parliament and of the Council as Regards Maximum Residue Levels for Imidacloprid in or on Certain Products. https://eur-lex.europa.eu/legal-content/EN/TXT/PDF/?uri=CELEX:32021R1881&from=EN.

[56] Tan G., Zhao Y., Wang M., Chen X., Wang B., Li Q.X. (2020). Ultrasensitive quantitation of imidacloprid in vegetables by colloidal gold and time-resolved fluorescent nanobead traced lateral flow immunoassays. Food Chem..

[57] Suryoprabowo S., Wu A., Liu L., Kuang H., Xu C., Guo L. (2023). A Rapid immunochromatographic method based on gold nanoparticles for the determination of imidacloprid on fruits and vegetables. Foods.

[58] Wang L., Cai J., Wang Y., Fang Q., Wang S., Cheng Q., Du D., Lin Y., Liu F. (2014). A bare-eye-based lateral flow immunoassay based on the use of gold nanoparticles for simultaneous detection of three pesticides. Microchim. Acta.

[59] Gao J., Zhang T., Fang Y., Zhao Y., Yang M., Zhao L., Li Y., Huang J., Zhu G., Guo Y. (2024). On-site rapid detection of multiple pesticide residues in tea leaves by lateral flow immunoassay. J. Pharm. Anal..

[60] Sheng W., Sun M., Bai D., Ren L., Ya T., Jin Z., Wang S., Wang Z., Tang X. (2024). Colorimetric and photothermal double-model immunochromatographic assay based on MoS2@Au nanocomposite for high sensitive detection of imidacloprid in food. Microchem. J..

[61] Cao H., Cao S., Han Y., Zhang W., Wei Z., Ye T., Yuan M., Yu J., Wu X., Hao L. (2022). Synthesis of quantum dot encoded multicolour nanobeads for the ultrasensitive and multiplex immunochromatographic detection of neonicotinoid insecticides. Sens. Actuators B Chem..

[62] Wang Y., Zhang M., Bu T., Bai F., Zhao S., Cao Y., He K., Wu H., Xi J., Wang L. (2023). Immunochromatographic Assay based on Sc-TCPP 3D MOF for the rapid detection of imidacloprid in food samples. Food Chem..

[63] Tursen J., Yang T., Bai L., Li D., Tan R. (2021). Determination of imidacloprid and acetamiprid in bottled juice by a new DLLME-HPLC. Environ. Sci. Pollut. Res..

[64] Yang Q., Ai X., Dong J., Liu Y., Zhou S., Yang Y., Xu N.A. (2021). QuEChERS-HPLC-MS/MS method with matrix matching calibration strategy for determination of imidacloprid and its metabolites in *Procambarus clarkii* (crayfish) tissues. Molecules.

[65] Harischandra Naik R., Ratnamma, Sangamesh V., Pallavi M.S., Saroja Rao N., Saraswati M., Pavankumar K., Arunkumar H., Bheemanna M., Prabhuraj A. (2023). Determination of imidacloprid in brinjal and okra fruits, decontamination and its dietary risk assessment. Heliyon.

[66] Erminia Schiano M., Sodano F., Cassiano C., Magli E., Seccia S., Grazia Rimoli M., Albrizio S. (2024). Monitoring of seven pesticide residues by LC-MS/MS in extra virgin olive oil samples and risk assessment for consumers. Food Chem..

[67] Harischandra N.R., Pallavi M.S., Bheemanna M., PavanKumar K., Chandra Sekhara Reddy V., Nidoni Udaykumar R., Paramasivam M., Yadav S. (2021). Simultaneous determination of 79 pesticides in pigeonpea grains using GC–MS/MS and LC–MS/MS. Food Chem..

[68] Al-Hawadi J.S., Al-Sayaydeh R.S., Al-Rawashdeh Z.B., Ayad J.Y. (2023). Monitoring of imidacloprid residues in fresh fruits and vegetables from the central parts of Jordan. Heliyon.

[69] Zhai R., Chen G., Liu G., Huang X., Xu X., Li L., Zhang Y., Xu D., Abd El-Aty A.M. (2023). Comparison of chemiluminescence enzyme immunoassay (Cl-ELISA) with colorimetric enzyme immunoassay (Co-ELISA) for imidacloprid detection in vegetables. Foods.

[70] You T., Ding Y., Huang Y., Lu Y., Wang M., Hua X. (2022). Identification and application of two promising peptide ligands for the immunodetection of imidacloprid residue. Foods.

