# Supplementary material for: Highly Sensitive Lateral Flow Immunodetection of the Insecticide Imidacloprid in Fruits and Berries Reached by Indirect Antibody–Label Coupling
Source: Foods. 2024 Dec 25;14(1):25. doi: 10.3390/foods14010025 (PMC11719783; doi:10.3390/foods14010025)
Supplement: Supplementary file 1 [file foods-14-00025-s001.zip › foods-3312907-supplementary.pdf]

Supplementary Information

Highly sensitive lateral flow immunodetection of insecticide imidacloprid in fruits and berries reached by indirect antibody-label coupling

Lyubov V. Barshevskaya, Elena A. Zvereva, Anatoly V. Zherdev, Boris B. Dzantiev*,

A.N. Bach Institute of Biochemistry, Research Center of Biotechnology of the Russian Academy of Sciences, Leninsky prospect 33, Moscow 119071, Russia

* Correspondence: dzantiev@inbi.ras.ru, Tel.: +7-495-954-31-42

Table 1. Conditions of chromatographic detection.

Time, min	A, vol %	B, vol %
0.0 - 2.0	98	2
2.0 - 3.0	98 – 2	2-98
3.0 - 7.0	2	98
7.0 – 7.5	2 – 98	98-2
7.5 - 12.0	98	2

A – 1% formic acid solution in water; B – acetonitrile.

Imidacloprid was identified by HPLC-MS/MS based on the absolute retention time of chromatographic peaks of target substance recorded in multiple reaction monitoring mode. Chromatogram of the sample containing imidacloprid at 25 ng/mL is shown in Figure S1.

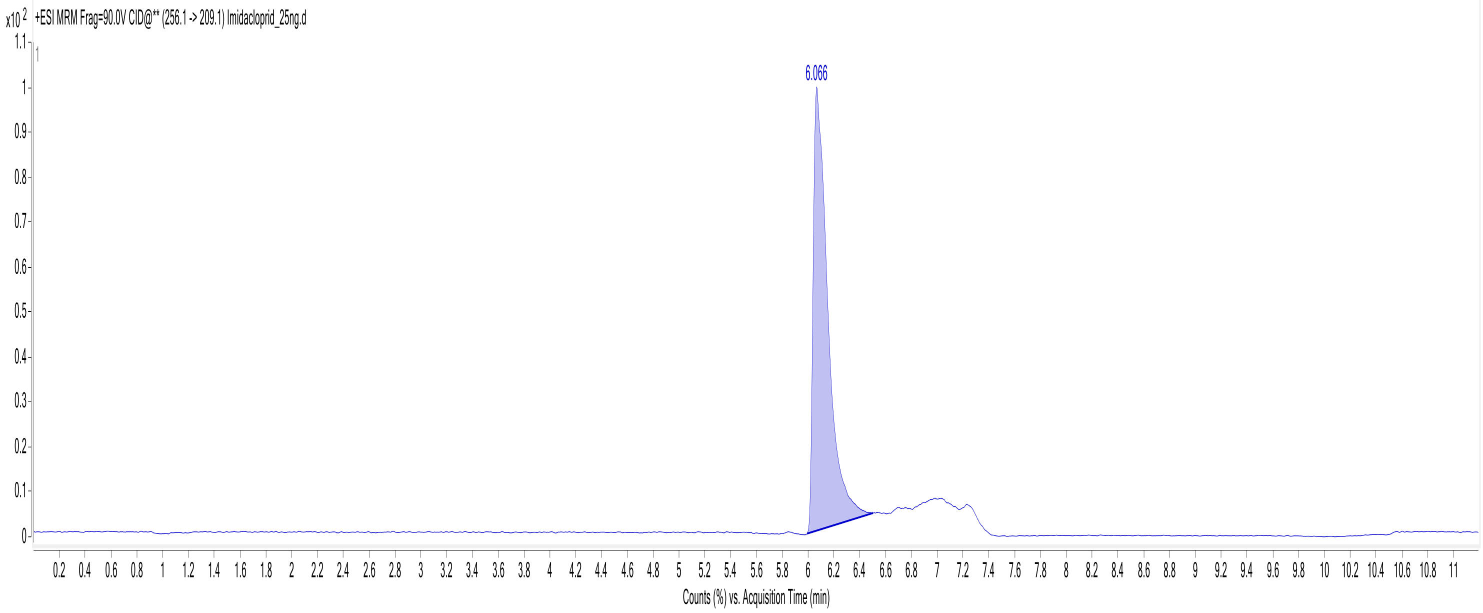

Figure S1. Chromatogram of the sample with imidacloprid at concentration of 25 ng/mL.

Calibration plot allows determining imidacloprid content in the range of 1000-0.1 ng/mL (Figure S2). Its regression equation is: y = 2.410442*x^2 + 6870.751152*x; R^2 = 0.99951227.

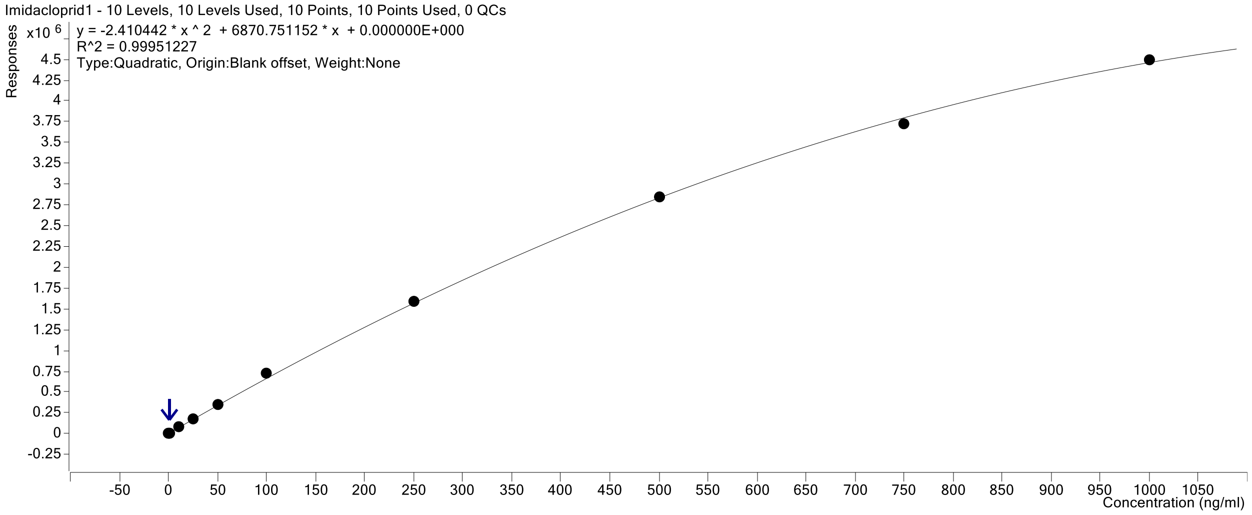

Figure S2. Linear calibration plot for imidacloprid.

Freshly squeezed juices preparations were first filtered and then used for HPLC-MS/MS. When analyzing orange, grape, and apple juice samples, the signal in the region of imidacloprid release (approximately 6 min) did not exceed the baseline fluctuations. Thus, the juice samples used did not contain significant concentrations of imidacloprid sufficient for detection and identification by HPLC-MS/MS (Figure S3). The detection limit of the method was 50 pg/mL.

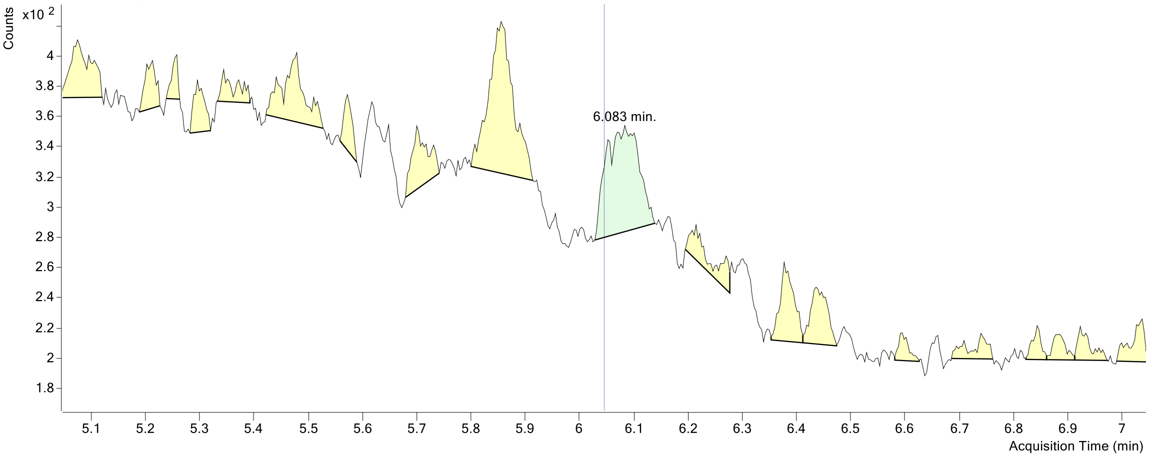

(a)

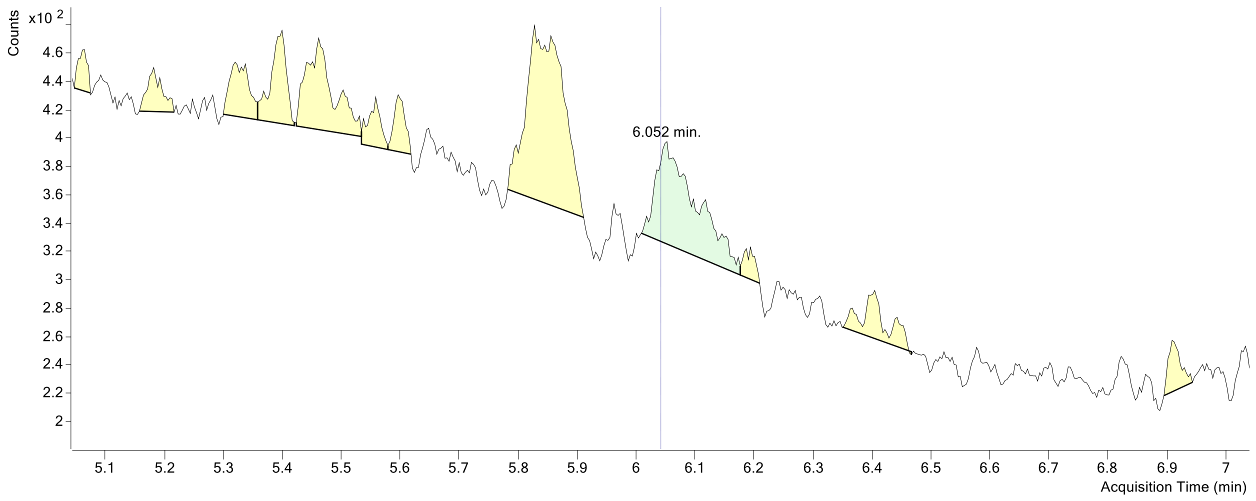

(b)

Figure S3. Example of chromatogram in grape juice (a) and orange juice (b).

To confirm the statement that the developed LFIA doesn't give any cross-reactions , we performed ELISA to determine non-neonicotinoid pesticides (fipronil, chlorpyrifos, cyhalothrin, cyproconazole and paraquat) using antibodies against imidacloprid. For this purpose, one of the specified pesticides at a concentration of 100-0.002 ng/mL and antibodies to imidacloprid at a concentration of 20 ng/mL were added microplate wells with adsorbed imidacloprid-BSA conjugate. According to the data obtained, no competitive interaction was observed between the added pesticide and the imidacloprid-BSA conjugate.

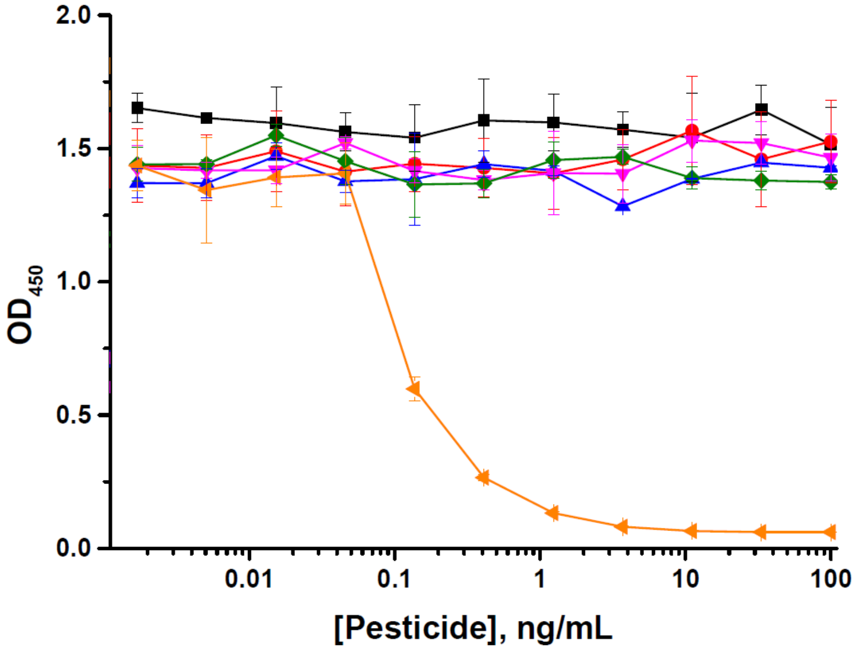

Figure S4. Selectivity testing of specific antibodies in ELISA. Black curve – paraquat; red curve – fipronil; blue curve – lambda-cyhalothrin; pink curve – chlorpyrifos; green curve – cyproconazole; orange curve – imidacloprid.
